# Inflammatory, Mechanical and Infectious Complications Associated with Peripheral Intravenous Catheters in Dogs and Cats: A Risk Factor Analysis

**DOI:** 10.3390/vetsci9030118

**Published:** 2022-03-06

**Authors:** Paolo Emidio Crisi, Francesca De Santis, Giovanni Aste, Pietro Giorgio Tiscar, Francesco Mosca, Agostina Gasparini, Andrea Felici, Laura Ferroni, Arianna Miglio, Morena Di Tommaso, Alessia Luciani

**Affiliations:** 1 Faculty of Veterinary Medicine, University of Teramo, località Piano d’Accio, Strada Provinciale 18, 64100 Teramo, Italy; fdesantis@unite.it (F.D.S.); gaste@unite.it (G.A.); pgtiscar@unite.it (P.G.T.); fmosca@unite.it (F.M.); gaspariniagostina@gmail.com (A.G.); mditommaso@unite.it (M.D.T.); aluciani@unite.it (A.L.); 2 Istituto Zooprofilattico Sperimentale dell’Umbria e Delle Marche-Togo Rosati (IZSUM), Via Salvemini 1, 06126 Perugia, Italy; a.felici@izsum.it (A.F.); l.ferroni@izsum.it (L.F.); 3 Department of Veterinary Medicine, University of Perugia, Via San Costanzo 4, 06126 Perugia, Italy; arianna.miglio@unipg.it

**Keywords:** catheters, complications, nosocomial infection, phlebitis

## Abstract

The placement of peripheral intravenous catheters (PIVC) is potentially associated with complications that negatively impact healthcare. Our study investigated factors associated with the occurrence of PIVC-related complications in dogs and cats at a Veterinary Teaching Hospital. The second aim was to determine the prevalence of PIVC bacterial colonization. A total of 76 dogs and 40 cats with PIVCs were evaluated for the occurrence of phlebitis and mechanical complications. The devices were removed when they ceased to be functional or when complications occurred, and the content was submitted for bacterial cultures and antimicrobial susceptibility tests. Both multivariable linear regression models and ROC analysis were employed. Complications were recorded in 46.6% of cases, and 20.7% of catheters yielded a positive culture. Among the isolates, 45% were classified as multi-resistant. In dogs, a ≥36-h indwelling time was associated with an increased risk of complications. Male cats seem more prone to developing complications, while the insertion of PIVCs under sedation may represent a protective factor in this species. In conclusion, PIVC-associated complications were frequently observed, and the high rate of positive culture for PIVCs, together with the presence of multi-resistant isolates, is a cause of concern in a hospital setting.

## 1. Introduction

The placement of short-term peripheral intravenous catheters (PIVC) is a common practice in veterinary medicine [1] and becomes an unavoidable tool for administering intravenous fluids or drugs [2]. Nevertheless, the use of intravenous devices is associated with potential mechanical, inflammatory, and infectious complications, which may negatively impact the welfare of patients or even outcomes in the most severe cases [3,4].

Catheter-related infections (CRI) in humans are a matter of concern, and despite the development of new devices such as antibiotic and antiseptic-coated catheters, preventive strategies remain the main goal [5]. Thus, the correct management of intravenous devices is important in limiting preventable complications; reducing the subsequent administration of antibiotics should also not be under-evaluated. Likewise, it is important to address mechanical complications as predisposing factors to infections and inflammatory complications, ultimately leading to prolonged hospitalization, increased morbidity and mortality, and a considerable increase in overall healthcare-associated costs [3,4,6].

There are many reports on scheduled catheter changes in human clinical practice to prevent complications such as thrombophlebitis. Early Centers for Disease Control and Prevention (CDC) recommends a replacement threshold from 48 to 72 h [7]. This threshold was subsequently prolonged up to 96 h, and recent studies have shown no benefit of routine replacement, suggesting that clinically indicated replacement is not only safe but also saves costs and avoids unnecessary painful procedures [8,9]. Studies conducted in veterinary hospitals indicated the association between nosocomial infections and contamination of intravenous catheters, with devices’ contamination rates ranging from 15.4% to 39.6% in dogs and cats [10,11,12,13,14]. However, although the indwelling time of intravenous catheters appears to be a risk factor for developing infectious complications, several studies have suggested that routine replacement of catheters every 72 h may not decrease the risk of catheter-associated infections [10,15]. Moreover, it is noteworthy that multi-drug-resistant bacteria are frequently involved in nosocomial infections [16], with pets regarded as potential reservoirs. For the foregoing reasons, it is clear that careful management of peripheral intravenous catheters is crucial to ensure patient comfort and reduce workplace risk in veterinary hospitals, promoting public health and leading to One Health implementation.

Therefore, our study aimed to evaluate the incidence of mechanical and inflammatory complications associated with short-term peripheral intravenous catheters and determine the incidence of colonization in intravenous devices for dogs and cats in a Veterinary Teaching Hospital setting. Moreover, our secondary aim was to determine risk factors associated with the onset of such complications and potential timing for device turnover.

## 2. Materials and Methods

Our prospective observational study was conducted at the Veterinary Teaching Hospital (VTH) of the Faculty of Veterinary Medicine of Teramo, Italy. Dogs and cats that required PIVCs were consecutively enrolled over a 6-month period. Signalment, the reason for admission, or final diagnosis were recorded for all patients.

All of the PIVCs were placed by the qualified medical staff of VTH. The hair over the insertion site was clipped, and the skin was disinfected using a standard protocol. Afterward, the operators classified the procedure as “easy” or “difficult” [1]. The animals’ reaction at the time of placement was scored using a simple descriptive scale as absent, slight, moderate, or strong [1]. The devices were fluorinated ethylene propylene catheters (FEP-Teflon; Smiths Medical Jelco, Tokyo, Japan); the indwelling catheter was secured with at least three pieces of adhesive tape and covered with a soft cotton bandage and an auto-adhesive elastic bandage.

The following information was collected for the animals: The insertion site (i.e., cephalic or saphenous access); the conditions of the insertion site, defined as good or poor depending on the quality of the clipped area, visibility, palpability, and stability of the vein [1]; the previous placement of other intravenous catheters in the same site; the reaction of the patient at insertion; and the number of attempts and size of the devices. If completed under sedation, the procedure was also recorded.

All drugs and fluids administered through the catheter were recorded, including antibiotics, irritants, and vesicants. Specifically, drugs capable of producing discomfort or pain from irritation in the internal lumen of the vein, with or without immediate external signs of vein inflammation, were considered irritants. Drugs capable of causing blisters, tissue sloughing, or necrosis after escaping into surrounding tissues were considered vesicants. Medications and solutions with extreme pH (≤5 or ≥9) or osmolarity (≥900 mOsm/L) and others encountered in the noncytotoxic vesicant list developed by the Infusion Nurse Society were included in these defined classes [17,18,19,20].

Every 12 h, the patients underwent a physical examination, and insertion sites were assessed for cleaning, patency, and functionality. Mechanical complications were defined as kinking, dislocation, occlusion of the device, and signs of infiltration or extravasation. The presence and extension of inflammation and early signs of phlebitis were evaluated using a Visual Infusion Phlebitis (VIP) grading scale from 0 to 5 [21]. Inflammatory complications were defined as the presence of pain, erythema, swelling, vein induration, or pyrexia identified by a VIP score of 2 or more [2,3]. When mechanical and inflammatory complications occurred simultaneously, they were classified as mixed.

Devices were removed when they ceased functioning or when inflammatory or mechanical complications occurred. The VIP score at the moment of removal, hours of permanence, and the reason for the devices’ removal were recorded.

Each device was removed, avoiding contamination, and the contents from each catheter were collected using the technique of turbulent flushing [22] in a sterile tube submitted to conventional bacterial cultures. Also, patients with systemic signs of inflammation/sepsis or true infections at the time of the device’s removal (i.e., depressed mental status, tachycardia, tachypnea, hypotension, hyperthermia, hypothermia, neutrophilia, and neutropenia) underwent blood culture. The threshold for semi-quantitative cultures of flushing fluid was five colony-forming units per milliliter (CFU/mL) [11]. Bacteria from pure cultures were identified with Gram staining, catalase, and oxidase tests; the identification at the species level was conducted through API^®^ systems (BioMérieux, Marcy-l’Etoile, France), including API 20E for Enterobacteriaceae, API 20NE for Gram-negative non-Enterobacteriaceae, and API 32 Staph for staphylococci, micrococci, and related genera. Antimicrobial susceptibility tests were performed on bacteria isolated from pure cultures according to the disk diffusion method, and the results were interpreted according to dog/cat breakpoints by the Clinical and Laboratory Standard Institute (CLSI. Performance Standards for Antimicrobial Disk and Dilution Susceptibility Tests for Bacteria Isolated from Animals. 5th ed. CLSI supplement VET01S, Clinical and Laboratory Standard Institute, 2020). If breakpoints were not available for specific antimicrobials, human breakpoints were applied (CLSI. Performance Standards for Antimicrobial Susceptibility Testing. 31st ed. CLSI supplement M100. 2021). The antibiotics used to determine antimicrobial resistance were: amoxi-clavulanic acid, amikacin, azithromycin, cefalexin, cefepime, ceftazidime, cefuroxime, enrofloxacin, cefazolin, trimethoprim-sulfamethoxazole, imipenem, and clindamycin. Strains resistant to at least three antimicrobial classes were defined as multi-resistant [23].

### Statistical Analysis

The statistical analyses of collected data were conducted separately in dogs and cats. After a preliminary descriptive assessment of the sample distribution, for questions that included more than two possible answers ranked in an ordinal scale, the answer of intermediate value was assigned either to the lower or higher category to obtain dichotomous variables with more balanced distributions. The indwelling time was considered both quantitative (number of hours) and qualitative with the categorization (T < 24 h; 24 ≤ T < 48 h; T ≥ 48 h). The VIP score was considered both quantitative (ranging from 0 to 5) and binary variables after three alternative categorizations (score 0 vs. score ≥ 1; score 0–1 vs. score ≥ 2; score 0–2 vs. score ≥ 3).

Multiple exploratory bivariate analyses were conducted to identify redundant variables, select candidate predictors for intravenous catheter-related complications, and choose an optimal response endpoint variable for the purpose of this study. Statistical associations for all dichotomous variables were investigated using the Fisher test on all possible contingency tables. We employed the Wilcoxon rank-sum test to assess statistical differences in age, weight, indwelling time, or VIP score across different groups of patients after assessing the normality of data distribution using the Shapiro–Wilk test. Additionally, we employed Chi-squared trend tests to assess whether response values (e.g., percentage of patients with a VIP score higher than a certain level) increase or decrease across the ordered groups (as indwelling time categories).

Then, the risk factor analysis for increasing VIP score was conducted using linear logistic regression models with a stepwise backward approach to select independent variables and build a model retaining only statistically significant factors. Associations previously found between the two variables were allowed to enter multivariable models separately.

In addition, indwelling time and a VIP score with three alternative categorizations were employed for an ROC analysis in canine patients to identify the optimal removal time of PIVCs, preventing the onset of phlebitis with a VIP score higher than a certain level.

Descriptive statistics were performed using Microsoft Excel 2013 (Microsoft Corporation) and Stata^®^ 16.1 statistical software (Special Edition; StataCorp LP, College Station, TX, USA). Bivariate analysis, risk factors analysis, and ROC analysis were conducted using Stata^®^. The Chi-squared test was conducted on the Epitools website (Sergeant, ESG, 2018. Epitools Epidemiological Calculators. Ausvet. http://epitools.ausvet.com.au. Accessed on 26 July 2021) setting a confidence level of 95%. The significance level was set at α = 0.05.

## 3. Results

A total of 116 patients were included in the study, including 40 cats and 76 dogs, from March to August 2018.

The feline population was entirely represented by domestic cats, whereas 46 (60.5%) dogs were mixed-breed and 30 (39.5%) were purebred. Characteristics of subjects included in the study are summarized in Table 1.

A total of 22 (55.0%) were referred for trauma, eight (20.0%) for respiratory signs, five (12.5%) were diagnosed with CKD, three (7.5%) had neurological signs, and two (5.0%) were accepted for elective ovariectomy.

A total of 26 (34.2%) were admitted at VTH for trauma, 14 (18.4%) for gastrointestinal disorders, eight (10.5%) for neurological signs, seven (9.2%) for endocrine disorders, five (6.5%) for elective orthopedic surgeries, and four (5.3%) for neoplastic disorders. Miscellaneous causes were reported in 12 cases (15.8%).

Schematic results regarding the insertion procedure of intravenous catheters, administration of antibiotics, or administration of irritants and/or vesicants are reported in Table 2. The substances administered and classified as irritants/vesicants were propofol, diazepam, midazolam, metoclopramide, maropitant, cefazolin, enrofloxacin, ampicillin-sulbactam, metronidazole, dextrose (12.5 and 25%), 0.45% sodium chloride, calcium gluconate, and amino acid solutions. 

In the feline population, 36 (90.0%) peripheral intravenous catheters were positioned in the cephalic vein, two (5.0%) in the lateral saphenous vein, and two (5.0%) in the medial saphenous vein. In dogs, 71 (93.4%) catheters were positioned in the cephalic vein, four (5.3%) in the lateral saphenous vein, and one (1.3%) in the medial saphenous vein.

Antibiotics were administered through a catheter in 21 cats (52.5%) and 23 dogs (30.3%). When the administration of antibiotics was compared among populations, a higher tendency was recorded in the feline population (*p* = 0.027) (Table 3).

**Figure 1:**
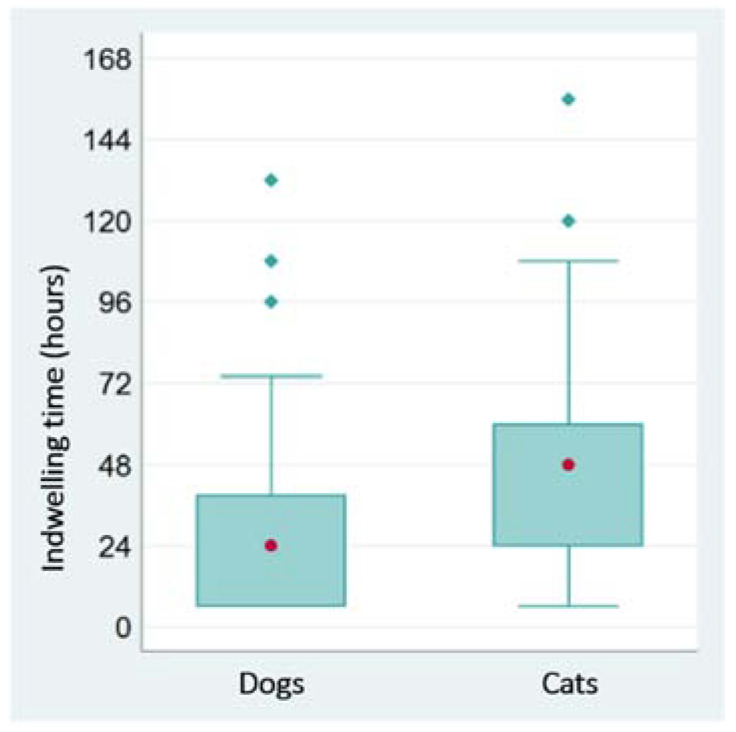
Boxplot of indwelling times of catheters in dogs and cats (red circles indicate the median values of the distribution).

The median time of permanence in indwelling catheters was 25.5 h (minimum 6 h; maximum 156 h). In dogs, the median permanence time of devices was 24 h (minimum 6 h; maximum 132 h), whereas, in cats, it was 48 h (minimum 6 h; maximum 156 h) (*p* = 0.0021) (Figure 1).

Variables of interest recorded at the time of catheter removal are summarized in Table 4. The devices were removed because of the onset of complications in 43% of dogs and 52% of cats. In the rest of the cases, the devices were removed because they were no longer needed. However, signs of phlebitis (i.e., VIP score ≥ 2) were observed in seven dogs and two cats, for which the reason for removing PIVC was categorized as disuse despite the evidence of phlebitis.

**Figure 2:**
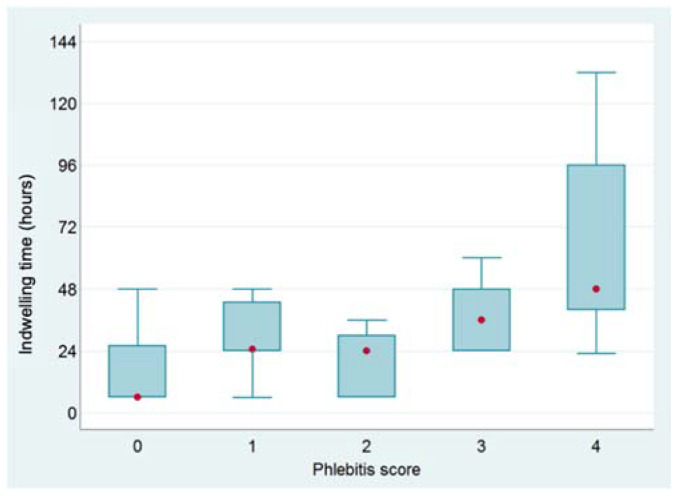
Box plot of indwelling time of catheters according to variations of phlebitis scores in dogs (red circles indicate the median values of the distributions).

**Figure 3:**
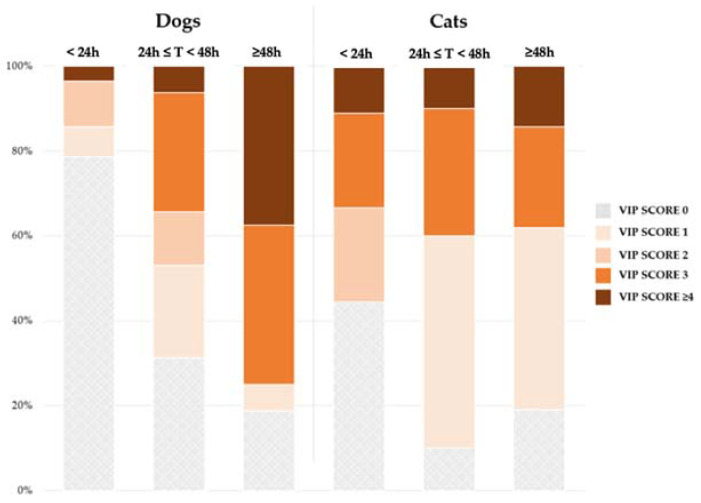
Distribution of the VIP scores according to the indwelling time of catheters. T: indwelling time; VIP: Visual Infusion Phlebitis.

**Figure 4:**
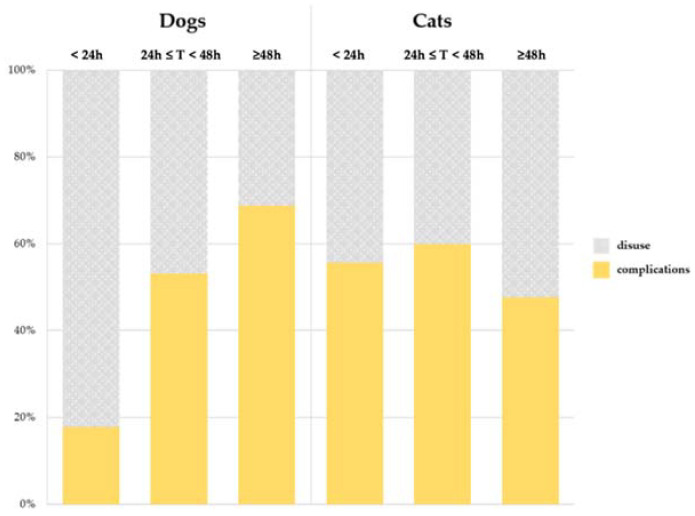
Causes of catheter removal according to the permanence time of the device.

In dogs, the indwelling time expanded across increasing values of VIP scores with a highly significant association (Wilcoxon: *p* < 0.001) (Figure 2). Additionally, using a different approach, the percentage of patients with a VIP score higher than a certain level (e.g., VIP score ≥3) increased as the duration of the device ranged between <24 h, at least 1 day but less than 48 h, and at least 48 h (Chi-squared: *p* < 0.0001) (Figure 3). On the contrary, no association between indwelling time and VIP score was found in cats. Similarly, the permanence of the device was positively related to the incidence of complications in dogs (Chi-squared: *p* = 0.0005). This association was not observed in cats (Figure 4).

**Figure 5:**
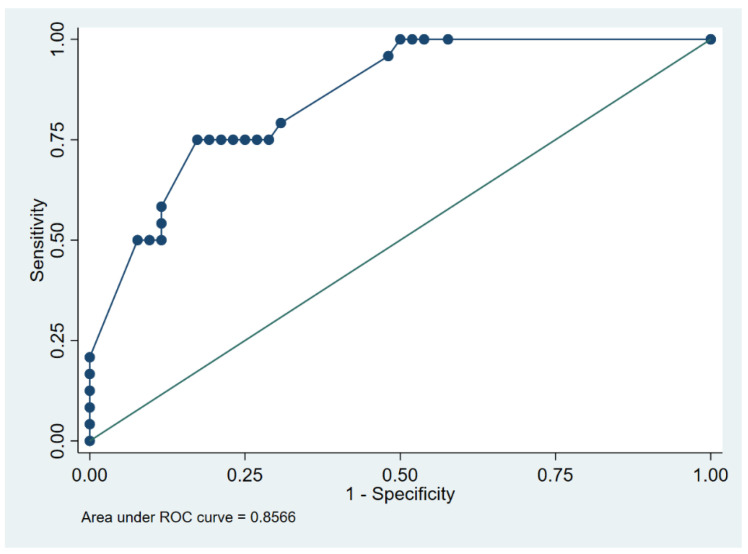
Receiver operating characteristic (ROC) curves determine the degree of phlebitis (absent–mild phlebitis vs. moderate–severe phlebitis) and indwelling time of catheters in dogs.

The multivariate analysis indicates that increased indwelling time in dogs (*p* < 0.001; R^2^ 0.38; slope 0.039, 95% CI 0.028–0.051), the male sex in cats (*p* = 0.009; R^2^ 0.31; slope 1.159, 95% CI 0.309–2.008), and the procedure conducted without sedation in cats (*p* = 0.001; R^2^ 0.31; slope 1.459, 95% CI 0.609–2.308) correlated with increased values of the VIP score. In dogs, the ROC curve for the discrimination between absent–mild phlebitis (VIP score 0–2) and moderate–severe phlebitis (VIP score ≥ 3) showed an AUC of 0.86 (95% CI 0.77–0.94) (Figure 5) with 36 h identified as the optimal cut-off (Table 5).

After the removal, 24/116 (20.7%) bacterial cultures from fluid obtained by catheter flushing scored positive; of them, eight (33.3%) from intravenous catheters were removed from cats and 16 (66.7%) from dogs.

No risk factors for contamination of intravenous devices were identified either in dogs or cats.

A total of 16 bacterial strains were isolated in dogs (*Pseudomonas aeruginosa n* = 6, *Escherichia coli n* = 3, *Staphylococcus Intermedius* Group (SIG) *n* = 3, *Serratia marcescens n* = 2, *Acinetobacter baumannii/calcoaceticus n* = 1, *Plesiomonas shigelloides n* = 1). Eight bacterial isolates were obtained in cats (*Enterobacter aerogenes n* = 3, *Enterobacter cloacae n* = 1, *Pseudomonas aeruginosa n* = 2, *Serratia marcescens n* = 1, *Acinetobacter baumannii/calcoaceticus n* = 1). The antimicrobial susceptibility test classified the isolates as multi-drug resistant (MDR) if they were resistant to three or more antimicrobial subclasses [24]. A total of 10 isolates (three from cats and seven from dogs) were classified as MDR (Table 6).

Three dogs admitted for trauma, parvoviral enteritis, and splenic hemangiosarcoma, respectively, underwent blood culture due to the presence of systemic signs of inflammation/infection at the time of catheter removal. One blood culture was negative in a dog with device positivity for *Pseudomonas aeruginosa*, while in two cases, bacterial growth was recorded. In particular, the blood culture of a dog affected by parvoviral enteritis with negative catheter culture showed a growth of a multiresistant strain of *Pseudomonas aeruginosa*, whereas the dog with splenic hemangiosarcoma and a device harboring *Psuedomonas aeruginosa* showed *Serratia marcescens* growth from blood culture.

## 4. Discussion

Peripheral intravenous catheter use is widespread in veterinary practice to administer fluids or medications. Nevertheless, PIVC use is generally associated with mechanical, inflammatory, and infectious complications [3,4]. Specifically, the rate of catheter-related infections is an important emerging issue in small animal veterinary medicine, mostly due to high morbidity, increased costs, and duration of treatment [14,24]. Furthermore, zoonotic risk arising from multi-drug resistant bacterial infections should be considered [25]. Infection control is a developed and recognized field in human medicine; however, in veterinary practice, it remains in its infancy, with few dedicated personnel, formal training opportunities, or coordinated surveillance programs [26,27].

Our study aimed to evaluate the incidence of complications and risk factors associated with using peripheral intravenous catheters in dogs and cats admitted to a Veterinary Teaching Hospital over a six-month period.

The incidence rate of non-infectious complications associated with peripheral intravenous catheters has only been evaluated in cats [28], and the analysis of risk factors associated with PIVC in veterinary medicine is still lacking.

The incidence of mechanical and inflammatory complications associated with the jugular venous catheter was evaluated by Adamantos et al., with complications observed in 39% of catheters, without differences between dogs and cats. Furthermore, most of these complications were mechanical (36/39 catheters), whereas inflammatory and infectious complications were less represented (10/39 catheters). Moreover, complications were more likely in patients requiring more than one attempt for placement and those that were ASA status 3–5; specifically, infectious complications were more likely in patients without general anesthesia for placement, those having medical rather than a surgical disease, and those placed out of theater [2].

Approximatively half of the devices, specifically 57% in dogs and 48% in cats, were removed because they were no longer needed. This data is slightly different compared with a previous study on the feline population, in which 78.6% of catheters were removed for disuse secondary to discharge from hospital or euthanasia [28].

Accordingly, the incidence of overall complications observed in the present study was notable compared with those reported in the aforementioned studies [2,28]. This discrepancy may be attributed to the different management of peripheral catheters in different settings. This study included dogs and cats with a wide variety of clinical conditions who were subsequently admitted as both intensive and non-intensive inpatients, whereas in other studies, only patients admitted in intensive care units were enrolled. Although strong and moderate reactions during catheter insertion were underrepresented, animals with a more active temperament might have disrupted the devices’ stability, increasing the likelihood of mechanical or mixed complications [28].

The median time of permanence in place of indwelling catheters was 25.5 h, with a median permanence time of devices significantly shorter in dogs (i.e., 24 h) than in cats (i.e., 48 h). In dogs, values of VIP scores increased as the indwelling time increased, and the length of permanence of devices was related to a high incidence of complications.

In studies conducted in dogs, a median indwell time of PIVCs ranging from 48 to 72 h was recorded [11,14,29], whereas the time of permanence in situ of PIVCs recorded in cats by Bush et al. was 28.8 h when devices were removed due to discharge, and 16.6 h when the withdrawal was consequent to other reasons; a further study found that PIVCs remained in place for a time >72 h in more than 80% of the included cats [12]. The permanence time of PIVCs recorded in dogs in our study was shorter than that reported in previous studies. This finding aligns with the higher incidence of complications, which may be a consequence of the heterogenicity of the population examined.

An optimal cut-off of 36 h for the replacement of PIVCs was identified in dogs. This value discriminates between absent-to-mild phlebitis (VIP score 0–2) and moderate-to-severe phlebitis (VIP score ≥ 3). On the other hand, there was no correlation between the permanence time of devices and VIP score values in the feline population.

In human medicine, several studies have been conducted to determine the impact of permanence time of PIVCs on the incidence of complications. Recent evidence suggests that the adoption of scheduled catheter change protocols was associated with a similar or higher risk of catheter-related complications [8,9,30]. However, according to current CDC guidelines, a threshold of 72–96 h is still suggested for replacement in adults. On the other hand, replacement is only recommended in children when clinically indicated, whereas in adults, it is still an unresolved issue [7]. Indwelling time was evaluated in veterinary medicine mainly as a risk factor for the onset of bacterial colonization; however, evidence about a routine replacement after a given period, rather than replacement when clinically indicated, is lacking. Specifically, catheters’ time of permanence in situ is related to contamination rates in devices and incidence of complications in some studies [11,14,29], whereas others failed to identify any association between these variables [12,13,28]. Interestingly, a correlation was found between the time of permanence and increasing values of VIP scores during the present study. The permanence time of PIVCs was not related to higher rates of bacterial colonization in devices. Furthermore, although a time of 48–72 h was evaluated as a possible risk factor in previous studies, the threshold of 36 h identified in our study was significantly shorter. This result may be attributed to the early onset of complications recorded in dogs and possibly to various clinical settings that included patients.

In cats, higher VIP scores were related to the male sex and the absence of sedation. In previous studies, sex was not identified as a risk factor for the onset of intravenous catheter-related complications in dogs and cats. A possible explanation for this finding may be related to the thicker skin of intact male cats. The thickness of feline skin ranges between 0.4 and 2 mm [31]; it is anecdotally reported that intact adult male cats have thicker skin, which impacts the insertion of the catheter with a potential reduction of device life. Unfortunately, no peer-reviewed evidence to support this claim was available.

Interestingly, procedures conducted under sedation appear to have a protective effect at the onset of complications in cats. This result is consistent with observations by a previous study on jugular intravenous catheters; however, the authors stated that a possible correlation with the ASA status of patients might have influenced this observation [2]. This finding may be related to the highest precision of insertion and securing of catheters ensured by sedation, especially in aggressive or frightened cats. Furthermore, since venipuncture can be a painful and stressful procedure, sedation is a valuable tool for minimizing pain and stress; thus, it should be considered in clinical practice to enhance the welfare of feline patients during hospitalization [32,33,34].

After the removal, 20.7% of bacterial cultures from PIVCs were positive, with an incidence of 20% in cats and 21% in dogs; this is largely consistent with previously reported contamination rates, ranging from 10.7 to 39.6% [10,11,12,13,14,29]. Although previous studies found higher rates of bacterial contamination in dogs [12,13], no association between species and positive bacterial cultures could be demonstrated [29].

*Enterobacter* spp. and *Pseudomonas* spp. were frequently isolated from cats, whereas *Pseudomonas* spp., *Staphylococcus* spp., and *Escherichia coli* were prevalent in dogs. *Enterobacteriaceae* and *E. coli* are usually recognized as agents of colonization for PIVCs in dogs and cats [11,12,29], possibly due to a high contamination rate of devices by enteric bacteria derived from saliva, feces, and urine [14]. Moreover, the skin of dogs and cats normally harbors *Staphylococcus* spp., which has also been implied as contaminating bacteria of intravenous catheters. In these cases, a faulty aseptic technique during catheter insertion or while manipulating the intravenous delivery system has been proposed as a possible explanation [29]. A further source of contamination is represented by healthcare personnel [27]. Healthcare workers’ inadequate hand hygiene has been identified as a direct risk factor for PIVC infection and the spread of infections in human hospitals [35,36].

Although a wide range of pathogens may be involved in nosocomial infections in veterinary facilities, currently, there is a strong focus on the emerging epidemic of multi-drug resistant bacteria due to dramatic increases in infections, limited antimicrobial options, and potential public health consequences. Specifically, *Acinetobacter* spp., extended-spectrum β-lactamase-producing *Enterobacteriaceae* (ESBLs), *Pseudomonas aeruginosa*, *Salmonella* spp., and multi-drug resistant *Staphylococcus aureus* (MRSA) have been classified as “serious antibiotic resistance threats” [27].

The overall incidence of multi-drug resistance recorded was notable, with a total of three multi-drug resistant isolates out of eight positive bacterial cultures in cats and seven out of sixteen in dogs, including *Enterobacter* spp., *Pseudomonas* spp., and *Staphylococcus* spp. Although the microbial colonization of intravenous devices may not be clinically relevant for patients, the high contamination rate by multi-drug resistant bacteria is a cause for concern because of risks related to zoonotic potential. Veterinary personnel and veterinary hospital environments are important key factors in the risk of gaining antibiotic-resistant pathogens by hospitalized dogs [24]. The hand hygiene of healthcare personnel has been recognized as the primary route of MRSA infection transmission in veterinary hospitals [24,37]. On the other hand, the threat of zoonoses arising from the exposition of veterinary healthcare personnel to multi-drug resistant bacteria should not be underestimated.

Overall, these findings strongly support the necessity of implementing infection-control programs in veterinary clinical settings.

## 5. Conclusions

PIVCs are frequently associated with mechanical, inflammatory, and mixed complications in veterinary clinical settings. Furthermore, nosocomial infections by multi-drug resistant bacteria that may contaminate intravenous devices are a cause for concern because of risks related to human health. In the present study, risk factors for the onset of catheter-related complications were assessed in dogs and cats in a veterinary hospital. In dogs, the permanence time of PIVCs was positively associated with increasing VIP scores. Consequently, an optimal cut-off of 36 h was identified as a replacement to minimize the severity of inflammatory complications. In cats, male sex and absence of sedation were identified as risk factors for higher VIP scores.

The contamination rate and frequency of isolation in multi-drug resistant bacteria were notable, raising concerns for human health risks and the spread of antimicrobial resistance.

## Figures and Tables

**Table 1:** Characteristics of sample population.

Species/Sex	Number of Subjects			Age (months)			Bodyweight (kg)		
	Total	Un-Neutered	Neutered	Median	Min	Max	Median	Min	Max
Dogs	76	63	13	60	2	144	14	1.5	55
Males	53	46	7	58	2	144	14.3	2	55
Females	23	17	6	66	3	144	13.6	1.5	25
Cats	40	29	11	54	4	144	3	1.5	5.5
Males	28	22	6	56	6	144	3.7	1.5	5.5
Females	12	7	5	49	4	132	2.9	1.9	4

**Table 2:** Results on the insertion procedure of intravenous catheters and administration of irritant and/or vesicants and antibiotics.

Variables		Dogs		Cats	
		N	%	N	%
Site of insertion	Cephalic vein	71	93%	36	90%
	Saphenous vein	5	7%	4	10%
Sedation	No	55	72%	28	70%
	Yes	21	28%	12	30%
Previous placement of PIVC at the same site	No	57	75%	28	70%
	Yes	19	25%	12	30%
Size of the device	26 Gauge	6	8%	2	5%
	24 Gauge	4	5%	12	30%
	22 Gauge	38	50%	23	58%
	20 Gauge	28	37%	3	8%
Number of attempts	1	67	88%	31	78%
	2	6	8%	4	10%
	3	3	4%	5	13%
Difficulty	Easy	69	91%	31	78%
	Difficult	7	9%	9	23%
Reaction	No	61	80%	31	78%
	Mild	9	12%	7	18%
	Moderate	6	8%	1	3%
	Strong	-	0%	1	3%
Site quality	Good	69	91%	35	88%
	Poor	7	9%	5	13%
Vesicants/irritants	No	37	49%	13	33%
	Yes	39	51%	27	68%
Antibiotics	No	53	70%	19	48%
	Yes	23	30%	21	53%

**Table 3:** Intravenous antibiotics administered in dogs and cats.

Classes	Molecules	Dogs (*n* = 23)	Cats (*n* = 21)
Penicillins	Ampicillin	3	1
Potentiated Penicillins	Ampicillin-sulbactam	3	10
Potentiated Penicillins + Fluoroquinolones	Ampicillin-sulbactam + Enrofloxacin	1	1
Potentiated Penicillins + Nitromidazoles	Ampicillin-sulbactam + Metronidazole	3	-
1st Generation Cephalosporins	Cefazolin	5	5
3rd Generation Cephalosporins	Ceftazidime	5	1
Fluoroquinolones	Enrofloxacin	3	2
Nitromidazoles	Metronidazole	-	1

**Table 4:** Variables at the time of the catheter removal.

Variables	Modalities	Dogs		Cats	
		N	%	N	%
Visual Infusion Phlebitis scale	Score 0	35	46%	9	23%
	Score 1	10	13%	14	35%
	Score 2	7	9%	2	5%
	Score 3	15	20%	10	25%
	Score 4	9	12%	5	13%
	Score 5	-	-	-	-
Onset of complications	No	43	57%	19	48%
	Yes	33	43%	21	52%
Complication/reason for removal	Disuse *	43	57%	19	48%
	Mechanic	9	12%	6	15%
	Inflammatory	7	9%	5	12%
	Mixed	17	22%	10	25%
Bacterial culture	Negative	60	79%	32	80%
	Positive	16	21%	8	20%

* intended as the primary reason for removal; this category includes 7 dogs and 2 cats which showed a VIP score ≥ 2.

**Table 5:** Sensitivity and specificity of different cut-off points to prevent the onset of moderate-to-severe phlebitis in dogs.

Cut-Point (h)	Sensitivity	Specificity	Classified	Youden’s J
(≥6)	100%	0%	31.6%	0.00
(≥12)	100%	42.3%	60.5%	0.42
(≥13)	100%	46.2%	63.2%	0.46
(≥20)	100%	48.1%	64.5%	0.48
(≥23)	100%	50.0%	65.8%	0.50
(≥24)	95.8%	51.9%	65.8%	0.48
(≥25)	79.2%	69.2%	72.4%	0.48
(≥26)	75.0%	71.2%	72.4%	0.46
(≥27)	75.0%	73.1%	73.7%	0.48
(≥29)	75.0%	75.0%	75.0%	0.50
(≥30)	75.0%	76.9%	76.3%	0.52
(≥32)	75.0%	78.9%	77.6%	0.54
(≥35)	75.0%	80.8%	79.0%	0.56
(≥36)	75.0%	82.7%	80.3%	0.58
(≥38)	58.3%	88.5%	79.0%	0.47
(≥40)	54.2%	88.5%	77.6%	0.43
(≥43)	50.0%	88.5%	76.3%	0.38
(≥47)	50.0%	90.4%	77.6%	0.40
(≥48)	50.0%	92.3%	79.0%	0.42
(≥60)	20.8%	100%	75.0%	0.21
(≥74)	16.7%	100%	73.7%	0.17
(≥96)	12.5%	100%	72.4%	0.13
(≥108)	8.3%	100%	71.1%	0.08
(≥132)	4.2%	100%	69.7%	0.04
(>132)	0%	100%	68.4%	0.00

**Table 6:** Phenotypic resistance profiles of multiresistant isolates.

Isolate	Species	PEN	AMI	MAC	CEF-1	CEF-2	CEF-3	CEF-4	FLUO	LINC	SXT	CARB	Tot. Classes R
*Escherichia coli*	D	R	R	S	-	R	R	S	R	-	S	-	5
*Escherichia coli*	D	R	R	S	-	R	R	-	R	-	S	R	6
*Enterobacter aerogenes*	C	R	S	S	-	R	S	S	S	-	S	R	3
*Enterobacter aerogenes*	C	R	S	S	-	R	S	S	S	-	S	R	3
*Enterobacter aerogenes*	C	R	S	S	-	R	S	S	S	-	S	R	3
*Plesiomonas shigelloides*	D	R	S	R	R	R	S	R	S	-	S	-	5
*Pseudomonas aeruginosa*	D	-	S	-	-	-	R	R	S	-	-	R	3
*Pseudomonas aeruginosa*	D	-	R	-	-	-	S	S	R	-	-	R	3
*Serratia marcescens*	D	S	R	S	R	S	S	S	S	-	R	-	3
*Staphylococcus Intermedius* Group	D	R	S	S	R	R	R	R	R	R	S	-	7

D, dog; C, cat; R, resistant to tested antimicrobial class; -, non-tested class; S, sensible; PEN, potentiated penicillin; AMI, aminoglycosides; MAC macrolides; CEF-k, cephalosporins, FLUO, fluoroquinolones; LINC, lincosamides, SXT potentiated sulfonamides; CARB, carbapenems.

## Data Availability

All study data are presented in the manuscript.
